# Tofacitinib treatment modulates the levels of several inflammation-related plasma proteins in rheumatoid arthritis and baseline levels of soluble biomarkers associate with the treatment response

**DOI:** 10.1093/cei/uxac085

**Published:** 2022-09-17

**Authors:** Atte Valli, Krista Kuuliala, Anniina Virtanen, Antti Kuuliala, Maaria Palmroth, Ritva Peltomaa, Krista-Liisa Vidqvist, Marjatta Leirisalo-Repo, Olli Silvennoinen, Pia Isomäki

**Affiliations:** Molecular Immunology Group, Faculty of Medicine and Health Technology, Tampere University, Tampere, Finland; Department of Bacteriology and Immunology, Faculty of Medicine, University of Helsinki, Helsinki, Finland; Molecular Immunology Group, Faculty of Medicine and Health Technology, Tampere University, Tampere, Finland; Department of Bacteriology and Immunology, Faculty of Medicine, University of Helsinki, Helsinki, Finland; Molecular Immunology Group, Faculty of Medicine and Health Technology, Tampere University, Tampere, Finland; Inflammation Center, Department of Rheumatology, Helsinki University Hospital and University of Helsinki, Helsinki, Finland; Centre for Rheumatic Diseases, Tampere University Hospital, Tampere, Finland; Department of Bacteriology and Immunology, Faculty of Medicine, University of Helsinki, Helsinki, Finland; Inflammation Center, Department of Rheumatology, Helsinki University Hospital and University of Helsinki, Helsinki, Finland; Molecular Immunology Group, Faculty of Medicine and Health Technology, Tampere University, Tampere, Finland; Fimlab Laboratories, Pirkanmaa Hospital District, Tampere, Tampere, Finland; Institute of Biotechnology, HiLIFE Helsinki Institute of Life Sciences, University of Helsinki, Helsinki, Finland; Molecular Immunology Group, Faculty of Medicine and Health Technology, Tampere University, Tampere, Finland; Centre for Rheumatic Diseases, Tampere University Hospital, Tampere, Finland

## Abstract

The data on the effects of tofacitinib on soluble proteins in patients with rheumatoid arthritis (RA) is currently very limited. We analyzed how tofacitinib treatment and thus inhibition of the Janus kinase—signal transducer and activation of transcription pathway affects the *in vivo* levels of inflammation-related plasma proteins in RA patients. In this study, 16 patients with active RA [28-joint disease activity score (DAS28) >3.2] despite treatment with conventional synthetic disease-modifying antirheumatic drugs (csDMARDs) started tofacitinib treatment 5 mg twice daily. Levels of 92 inflammation-related plasma proteins were determined by proximity extension assay at baseline and at 3 months. Tofacitinib treatment for 3 months, in csDMARD background, decreased the mean DAS28 from 4.4 to 2.6 (*P* < 0.001). Marked (>20%) and statistically significant (*P* < 0.05) changes were found in the levels of 21 proteins, 18 of which decreased and 3 increased. Of these proteins, 17 are directly involved in inflammatory responses or in the cellular response to cytokines. The highest (>50%) decrease was observed for interleukin-6 (IL-6), C-X-C motif chemokine ligand 1, matrix metalloproteinase-1, and AXIN1. Higher baseline levels of IL-6 and lower levels of C-C motif chemokine 11 and Delta and Notch-like epidermal growth factor-related receptors were associated with DAS28 improvement. Our results indicate that tofacitinib downregulates several proinflammatory plasma proteins that may contribute to the clinical efficacy of tofacitinib. In addition, soluble biomarkers may predict the treatment response to tofacitinib.

## Teaser

Sixteen patients with active rheumatoid arthritis, despite conventional synthetic disease-modifying antirheumatic drugs, started additional tofacitinib treatment. Blood samples were drawn at baseline and at 3-month follow-up visits, and plasma levels of 92 inflammation-related proteins were measured with proximity extension assay. Levels of 21 proteins changed substantially (>20%) and statistically significantly upon tofacitinib treatment, and the baseline levels of interleukin-6, C-C motif chemokine 11 and Delta and Notch-like epidermal growth factor-related receptor associated with the treatment response.

## Graphical Abstract

**Graphical Abstract:**
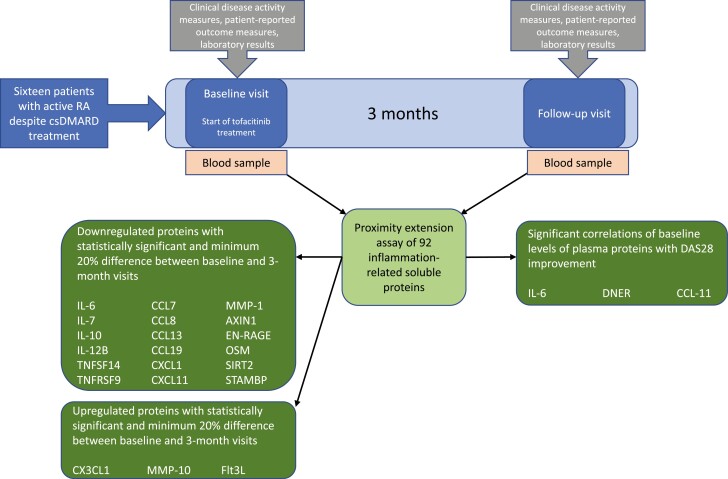

## Introduction

In rheumatoid arthritis (RA) various immune cells accumulate in the joints, a process partly driven by chemokines. Activated immune cells in the synovium communicate with each other and with synovial cells by direct cell-cell contact, as well as by secreting soluble mediators such as cytokines. In addition, matrix-degrading enzymes, such as matrix metalloproteinases (MMPs), are secreted, ultimately leading to the degradation of articular cartilage and bone [1]. Central cytokines regulating immune responses in RA include interferons (IFNs), Interleukin-1 (IL-1), IL-6, IL-7, IL-12, IL-15, IL-17, IL-23, tumor necrosis factor-α (TNF) and granulocyte-macrophage colony-stimulating factor [2].

The majority of the cytokines involved in RA act through the JAK-STAT signaling pathway that consists of Janus kinases (JAKs) and signal transducers and activators of transcription (STAT) [2]. A cytokine initiates the signaling pathway by binding to its receptor, resulting in JAK phosphorylation. The activated JAKs then induce STAT phosphorylation and dimerization, and subsequent translocation into the nucleus to regulate the expression of target genes [3]. We and others have shown that JAK-STAT pathways are constitutively active in circulating leukocytes of patients with RA [4–7]. Tofacitinib, the first JAK inhibitor developed for the treatment of RA, inhibits JAK1, JAK3, and to a slightly lesser extent JAK2 [8, 9]. Tofacitinib in a methotrexate background was shown to have similar efficacy compared to TNF inhibitor adalimumab in RA patients with active disease despite conventional synthetic disease-modifying drug (csDMARD) treatment [10].

Although the clinical effects of tofacitinib in RA have been well documented, the cellular effects of tofacitinib in RA patients *in vivo* have been less studied. For example, the knowledge of the effects of tofacitinib on cytokine or chemokine expression is mainly based on *in vitro* studies or animal models of arthritis. Tofacitinib has previously been shown to inhibit IL-17 and IFN-γ production and T cell activation *in vitro* by synovial and peripheral blood T cells of RA patients [11, 12]. In addition, tofacitinib suppressed the expression of IL-6, C-X-C motif chemokine ligand 9 (CXCL9), CXCL10, and CXCL11 in TNF-stimulated RA synovial macrophages [13], and expression of IL-6 in oncostatin-stimulated and the expression of C–C motif chemokine ligand 2 (CCL2), CCL5, and CXCL10 in TNF-stimulated RA fibroblast-like synoviocytes [14, 15]. Studies using immunodeficient mice with RA patient synovium and cartilage implants demonstrated that tofacitinib treatment resulted in decreased serum levels of human IL-6 and IL-8 [11]. Also, administration of tofacitinib downregulated the plasma levels of IL-6, granulocyte colony-stimulating factor, CCL2, and CXCL10 in mice immunized to develop collagen-induced arthritis [16].

Data regarding the effects of tofacitinib treatment on the levels of soluble proteins in RA is currently limited, and effects on only selected proteins have been reported. Tofacitinib treatment in RA patients *in vivo* has been shown to decrease the levels of circulating IL-6, CXCL10, MMP-3, and gliostatin [17–19]. Tofacitinib also reduced the expression of MMP-1, MMP-3, CCL2, CXCL10, and CXCL13 in synovial biopsies of RA patients with an inadequate response to methotrexate [19]. While tofacitinib has usually been observed to decrease the levels of immunological markers, an increase has been described, for example, for circulating levels of osteoprotegerin, osteocalcin, and vitamin D_3_ during six and 12 months of tofacitinib use in patients with RA [20]. The finding was assumed to reflect stabilization of bone density and positive balance of bone turnover by tofacitinib. Owing to the rarity of *in vivo* studies on tofacitinib use, novel markers upregulated by tofacitinib may be unidentified yet.

Studying the *in vivo* effects of tofacitinib helps us to understand the mechanisms of action of tofacitinib in RA patients. We have previously shown that tofacitinib inhibits multiple JAK-STAT pathways in a cytokine and cell population specific manner, and downregulates the expression of several JAK-STAT pathway components, in circulating leukocytes of RA patients [21]. In the current study, we have extended the analysis of tofacitinib treatment on the expression of a broad range of soluble inflammation-associated proteins in RA patients. The association between the levels of these proteins and tofacitinib treatment response was additionally studied.

## Patients and methods

### Patients

Patients were recruited from two rheumatology outpatient clinics (Tampere and Helsinki University Hospitals) between 2018 and 2020. Eligible patients were required to fulfill the 2010 ACR/EULAR classification criteria for RA and have an active disease at baseline visit: the Disease Activity Score for 28 joints based on C-reactive protein level (DAS28-4[CRP]) >3.2 despite treatment with csDMARDs. Key exclusion criteria were prior or current treatment with biologics or JAK inhibitors, a current infection, malignancy, severe hepatic impairment, pregnancy or lactation, hemoglobin <90 mg/dl, a neutrophil count of <1.9 × 10^9^/l, or a lymphocyte count <0.75 × 10^9^/l.

The study was approved by the National Committee on Medical Research Ethics (TUKIJA) and the Finnish Medicines Agency Fimea. The study was conducted according to the principles of the Declaration of Helsinki and Good Clinical Practice Guidelines. All patients gave written informed consent for the study.

### Study design

The study consisted of three visits: a screening visit 0–3 months before the baseline visit, the baseline visit, and a follow-up visit after 3 months of tofacitinib treatment. From the baseline visit on, the patients were treated with tofacitinib 5 mg twice daily throughout the study time. Ongoing csDMARD therapy and prednisolone (0–10 mg/day) were continued at stable doses. Patient-reported outcomes and clinical assessments were recorded at study visits. Treatment response was determined by the change in DAS28-4[CRP] between baseline and 3-month visits. At baseline and follow-up visits, whole blood samples were drawn into EDTA (Ethylene Diamine Tetra Acetic acid) tubes and kept on ice. Within an hour from sampling, plasma was isolated by centrifugation at 2000 *g* for 10 min at +4°C and subsequently stored at −80°C.

### Proximity extension assay for plasma proteins

Plasma samples from the baseline and follow-up visits were sent to Olink Bioscience AB (Uppsala, Sweden) for high-throughput analysis of 92 inflammation-associated proteins in proximity extension assay in 96-well plate format [22, 23].

The proximity extension assay is a dual-recognition immunoassay, where two matched antibodies labeled with unique DNA oligonucleotides simultaneously bind to a target protein in solution. This allows the two antibodies to converge and their DNA oligonucleotides to hybridize, serving as a template for a DNA polymerase-dependent extension step. This creates a double-stranded DNA “barcode” which is unique for the specific protein. The hybridization and extension are followed by PCR amplification. Finally, the concentration of the PCR product is directly proportional to the initial concentration of the target protein.

Relative levels of proteins were reported on an arbitrary Log_2_-based NPX (normalized protein expression) scale that does not allow absolute protein quantities to be compared across different proteins. The logarithmic scale was converted into a linear scale for further analysis and proteins were categorized according to the public access bioinformatics databases Uniprot, Human Protein Atlas, Gene Ontology, and DisGeNET.

### Statistical methods

Plasma proteins with baseline levels below the limit of detection (LOD) in more than 25% of the samples were discarded from further analysis. For the remaining proteins, the values below LOD were changed to equal LOD, as suggested by Olink. Wilcoxon signed-rank test was used to assess significance in DAS28 improvement and levels of soluble plasma proteins before and after tofacitinib treatment. *P*-values < 0.05 were considered statistically significant. Multiple comparisons were taken into account by considering only proteins that changed by more than 20% to be markedly affected by tofacitinib treatment. Spearman rank correlation was used to evaluate correlations between protein levels and clinical improvement. Confidence intervals were calculated with bootstrapping. Statistical analysis was carried out using SPSS (version 26; IBM, Armonk, NY, USA).

## Results

### Patients and clinical response to tofacitinib

Patient characteristics and the clinical response to tofacitinib treatment in the patient population have been previously reported in detail [21]. The study population consisted of 16 RA patients whose demographic and clinical characteristics are described in Table 1. Twelve (75%) patients received methotrexate (MTX) as part of their csDMARD regimen.

**Table 1:** Demographic and clinical characteristics of the patients (*n* = 16)

Age, years, mean (range)	58.4 (36.4–72.9)
Sex, female, *n* (%)	11 (69%)
Disease duration, years, mean (range)	9.6 (0.5–48.0)
Erosive disease, *n* (%)	7 (44%)
Rheumatoid factor positive, *n* (%)	11 (69%)
CCP-antibody positive, *n* (%)	12 (75%)
Disease activity (DAS28-4 [CRP]), ­median (IQR)	4.4 (3.6–4.9)
Swollen joint count, 0–46, median (IQR)	7 (6–9)
Tender joint count, 0–46, median (IQR)	11 (5–17)
Patient’s global assessment, VAS, 0–100 mm, median (IQR)	35 (31–46)
General health, VAS, 0–100 mm, median (IQR)	51 (37–65)
Pain, VAS, 0-100 mm, median (IQR)	43 (22–64)
HAQ disability index, 0–3, median (IQR)	0.813 (0.625–1.253)
csDMARD treatment, *n* (%)	
Single	3 (19%)
Double	7 (44%)
Triple	6 (37%)
Low-dose prednisolone, *n* (%)	8 (50%)

Cyclic citrullinated peptide (CCP); disease activity score for 28 joints (DAS28); interquartile range (IQR); visual analogue scale (VAS); health assessment questionnaire (HAQ); conventional synthetic disease-modifying antirheumatic drug (csDMARD).

According to DAS28-4 [CRP], 13 (81%) patients had moderate disease activity and 3 (19%) had high disease activity before treatment. During tofacitinib treatment the median DAS28 decreased from 4.4 to 2.6 (*P* < 0.001). At the follow-up visit, nine patients (56%) were in DAS28 remission, and four (25%) patients had low, two (13%) had moderate and one (6%) had high disease activity.

### Effects of tofacitinib treatment on inflammation-associated plasma proteins

Levels of 92 soluble plasma proteins were evaluated before and after 3-month tofacitinib treatment in csDMARD background. Proteins for which a significant proportion (>25%) of samples were under LOD at baseline were discarded from further analysis (Supplementary Table S1). Of the remaining 75 proteins, 22 were significantly downregulated and 10 were upregulated during tofacitinib treatment (Supplementary Table S1).

To gain better insight into clinically meaningful changes in protein levels during tofacitinib treatment, the percentage difference between protein levels before and after treatment were calculated. Table 2 shows levels of soluble plasma proteins before and after tofacitinib treatment for the 21 proteins that demonstrated both substantial (>20%) and statistically significant change during the treatment. Of these, the levels of 18 (86%) proteins decreased and 3 (14%) increased (Table 2, Supplementary Fig. S1). IL-6, CXCL1, MMP-1, and AXIN1 were most strongly affected and demonstrated a more than a 50% decrease between baseline and 3-month values.

**Table 2:** The levels of soluble plasma proteins in patients with rheumatoid arthritis before and after 3-month treatment with tofacitinib and csDMARDs. Proteins with statistically significant and minimum 20% difference between baseline and 3-month visits are shown. Results with at least 50% difference are shown in bold.

Protein	Before, mean (CI)	After 3 months, mean (CI)	Percentage difference (CI)	*P*
IL-6	34.2 (15.3–63.5)	11.2 (5.7–20.2)	**−67 (−1.2 to −159)**	0.004
IL-7	3.5 (3–4.1)	2.7 (2.3–3.2)	−24 (−10 to −40)	0.008
IL-10	21.7 (16.9–26.9)	16.1 (13.1–19.5)	−26 (−3 to −45)	0.025
IL-12B	73.6 (58.1–88.9)	57.4 (47.3–68.5)	−22 (−4.4 to −38)	0.039
TNFSF14	21.9 (18.9–25.2)	16.6 (14.2–19.2)	−24 (−11 to −36)	0.002
TNFRSF9	114 (91–140)	89.3 (73.2–106)	−22 (−9.8 to −35)	0.006
CCL7	4.2 (3.2–5.7)	2.8 (2.5–3.3)	−33 (−9.6 to −64)	0.016
CCL8	280 (205–374)	210 (167–256)	−25 (−9.9 to −44)	0.003
CCL13	11 755 (8664–15544)	7159 (6198–8198)	−39 (−16 to −66)	0.002
CCL19	581 (480–688)	377 (315–440)	−35 (−22 to −50)	<0.001
CXCL1	239 (158–325)	113 (93.4–134)	**−53 (−22 to −85)**	0.002
CXCL11	162 (115–215)	109 (76.8-153)	−33 (5.5 to −70)	0.005
CX3CL1	16.4 (14.8–18.1)	19.7 (17.3–22.6)	20 (7.2 to 35)	0.011
MMP-1	25 120 (15 059–37423)	9175 (6053–13,413)	**−63 (−24 to −111)**	0.013
MMP-10	483 (403–576)	645 (482–873)	34 (10 to 61)	0.018
AXIN1	14.6 (9.4–20.8)	6.6 (4.9–8.2)	**−55 (−20 to −98)**	0.006
EN-RAGE	19.4 (12.8–26.6)	11.9 (8.1–16.2)	−38 (−14 to −64)	0.005
Flt3L	663 (583–734)	832 (717–945)	26 (9.3 to 42)	0.003
OSM	27.9 (18.5–41.2)	17.3 (12.5–23.6)	−38 (−16 to −66)	0.002
SIRT2	16 (11.1–21.9)	9.5 (7.6–11.7)	−41 (−15 to −74)	0.006
STAMBP	32.4 (25.5–40.8)	21.4 (18.5–24.8)	−34 (−14 to −59)	0.003

Wilcoxon signed-rank test (*P*). Confidence intervals (CI) were calculated with bootstrapping. Conventional synthetic disease-modifying antirheumatic drug (csDMARD); interleukin (IL); Tumor necrosis factor superfamily member 14 (TNFSF14); tumor necrosis factor receptor superfamily member 9 (TNFRSF9); C–C motif chemokine (CCL); C-X-C motif chemokine (CXCL); C-X3-C motif chemokine (CX3CL); matrix metalloproteinase (MMP); S100 calcium-binding protein A12 (EN-RAGE); FMS-like tyrosine kinase 3 ligand (Flt3L); oncostatin M (OSM); NAD-dependent deacetylase sirtuin 2 (SIRT2); STAM-binding protein (STAMBP).

The proteins that were significantly affected by tofacitinib treatment included several cytokines and chemokines as well as two matrix metalloproteinases (MMP-1 and MMP-10). To gain better insight into the function of these proteins, they were grouped based on their involvement in biological processes (Table 3). The tofacitinib-affected proteins most frequently had a role in processes of cellular response to cytokine stimulus (*n* = 15) and inflammatory response (*n* = 14), and the least frequently in processes of extracellular matrix organization (*n* = 2) and response to hypoxia (*n* = 1). Additionally, the protein–protein interaction network was visually analyzed by STRING (Search Tool for the Retrieval of Interacting Genes/Proteins; Supplementary Supplementary Fig. S2). Strongest evidence for interactions (confidence score > 0.9) was demonstrated for proteins IL-6, IL-10, IL-12B, CCL19, CXCL1, MMP-1, MMP-10, and oncostatin M (OSM).

**Table 3:** The involvement of soluble plasma proteins in different biological processes categorized according to the public access bioinformatics databases Uniprot, human protein atlas, gene ontology, and DisGeNET. Proteins with statistically significant and minimum 20% difference during tofacitinib treatment are shown. Eighteen proteins that decreased during tofacitinib treatment are shown on the left, and three proteins that increased on the right.

Biological process	IL-6	IL-7	IL-10	IL-12B	TNFSF14	TNFRSF9	CCL7	CCL8	CCL13	CCL19	CXCL1	CXCL11	MMP-1	AXIN1	EN-RAGE	OSM	SIRT2	STAMBP	CX3CL1	MMP-10	Flt3L	Count
Cellular response to cytokine stimulus	X	X	X	X	X	X	X	X	X	X	X	X	X			X			X			15
Inflammatory response	X		X	X		X	X	X	X	X	X	X		X	X	X			X			14
Apoptotic process	X	X	X	X	X	X				X				X			X	X	X		X	12
Chemotaxis	X		X		X		X	X	X	X	X	X			X				X			11
MAPK cascade	X						X	X	X	X					X	X			X		X	9
Secretion	X		X			X		X		X	X				X	X			X			9
Cell adhesion	X	X	X	X	X					X									X		X	8
Cell activation in immune ­response	X		X	X						X	X				X							6
Regulation of immune response	X		X	X						X												4
Extracellular matrix ­organization													X							X		2
Response to hypoxia																	X					1

Mitogen-activated protein kinase (MAPK); interleukin (IL); tumor necrosis factor superfamily member 14 (TNFSF14); tumor necrosis factor receptor superfamily member 9 (TNFRSF9); C–C motif chemokine (CCL); C-X-C motif chemokine ligand (CXCL); C-X3-C motif chemokine ligand (CX3CL); matrix metalloproteinase (MMP);S100 calcium-binding protein A12 (EN-RAGE); Oncostatin M (OSM); NAD-dependent deacetylase sirtuin 2 (SIRT2); STAM-binding protein (STAMBP); FMS-like tyrosine kinase 3 ligand (Flt3L).

We also investigated whether the changes in protein levels during tofacitinib treatment were associated with treatment response. Of the 21 proteins studied, changes in IL-6 and tumor necrosis factor superfamily member 14 (TNFSF14) levels significantly correlated with treatment response (Table 4, right panel).

**Table 4:** Significant correlations of baseline levels of plasma proteins (left panel) or changes in plasma protein levels (right panel) with improvement in DAS28-4[CRP] during 3-month treatment with tofacitinib and csDMARDs. Correlations for a change in protein levels were analyzed only for proteins that showed statistically significant (*P* < 0.05) and minimum 20% difference by tofacitinib treatment.

Protein				
	Level at baseline		Decrease during treatment	
	*r*	*P*	*r*	*P*
IL-6	0.612 (0.141–0.899)	0.012	0.639 (0.106–0.918)	0.008
TNFSF14			0.524 (0.027–0.867)	0.037
CCL-11	−0.553 (−0.845 to 0.066)	0.026		
DNER	−0.514 (−0.847 to 0.063)	0.042		

Spearman correlation coefficients (*r*) and confidence intervals (CI) calculated with bootstrapping are shown. Empty spaces denote correlations that were not significant (*P* > 0.05). Composite disease activity score for 28 joints based on C-reactive protein level (DAS28-4[CRP]); conventional systemic disease-modifying antirheumatic drug (csDMARD); interleukin (IL); tumor necrosis factor superfamily member 14 (TNFSF14); C-C motif chemokine 11 (CCL-11); delta and Notch-like epidermal growth factor-related receptor (DNER).

Finally, we also investigated whether the levels of proteins that were markedly affected by tofacitinib treatment correlated with disease activity at baseline. We found no significant associations between disease activity and proteins levels at baseline or the change in protein levels during tofacitinib treatment (Supplementary Table S2)

### Baseline levels of plasma proteins correlate with treatment response

To investigate whether baseline levels of soluble proteins are associated with treatment response to tofacitinib, correlation coefficients were calculated between protein levels at baseline and DAS28 improvement during tofacitinib treatment. The baseline level of IL-6 correlated positively with DAS28 improvement, whereas CCL11 and Delta and Notch-like epidermal growth factor-related receptor (DNER) showed a negative correlation (Table 4, left panel).

## Discussion

The results show that plasma levels of several inflammation-associated proteins are altered by the JAK inhibitor tofacitinib *in vivo*, as determined after 3 months of tofacitinib treatment in patients with csDMARD-irresponsive RA. Of 92 proteins determined, 18 showed a decrease of over 20%, and four of these (IL-6, CXCL1, MMP-1, and AXIN1) showed a >50% decrease. Only three proteins were increased (>20%) during tofacitinib treatment: C-X3-C motif chemokine ligand 1 (CX3CL1), MMP-10, and FMS-like tyrosine kinase 3 ligand (Flt3L). Treatment response correlated positively with baseline IL-6 levels, and negatively with baseline CCL11 and DNER levels.

To the best of our knowledge, the current study is the first to investigate the effects of tofacitinib on such a large range of inflammation-associated plasma proteins in RA patients *in vivo.* Previous *in vitro* studies have shown tofacitinib to suppress the production of IL-6, IL-17, IFN-γ, CCL2, CXCL9, CXCL10, and CXCL11 by RA T cells, macrophages, and fibroblasts [11–15]. Also, in newly discovered pathogenic dendritic cells in the circulation of RA patients, tofacitinib suppressed the production of IL-1β, IL-8, and IL-13 significantly, and that of IL-6 non-significantly [24]. In animal models of arthritis tofacitinib treatment has led to decreased levels of circulating IL-6, IL-8, CCL2, and CXCL10 [11, 16]. Our *in vivo* results confirmed that IL-6 and CXCL11 are significantly downregulated in RA patients treated with tofacitinib, while IL-8, IL-17, IFN-γ, CCL2, CXCL9, and CXCL10 were not affected. These differences highlight the importance of studying the biochemical effects of therapies such as JAK inhibitors in RA patients *in vivo*.

A recent *in* vivo study shows that tofacitinib 10 mg twice daily in MTX background downregulates the expression of MMP-1, MMP-3, CCL2, CXCL10, and CXCL13 in the synovium and the plasma levels of CXCL10 in RA patients during 28-day treatment [19]. We demonstrate here that while plasma MMP-1 levels were downregulated, levels of CCL2 or CXCL10 were not affected by 3-month tofacitinib 5mg twice daily plus csDMARD treatment *in vivo*. It is plausible that the effects of tofacitinib on the expression of chemokines in synovial tissue are not directly comparable to the effects of tofacitinib on circulating chemokine levels. The different result concerning plasma CXCL10 levels may be due to the differences in the treatment duration (1 month versus 3 months), tofacitinib dose (10 versus 5 mg twice daily) or patient characteristics between the study of Boyle et al. and the current study.

In accordance with our finding, it has been previously observed that tofacitinib treatment leads to a marked decrease in circulating IL-6 levels in RA patients [17] and in animal models of arthritis [11, 16]. Therefore, tofacitinib seems to have profound suppressive effect on IL-6 production in arthritis. A decrease in plasma IL-6 levels during treatment of active RA has also been described for MTX [25] and for the combination of MTX and TNF inhibitor [26]. On the contrary, the anti-IL-6 receptor antibody tocilizumab significantly increases plasma IL-6 levels, but on the other hand, it also induces a huge increase in soluble IL-6 receptor levels which counteracts the biological effect of IL-6 [27]. Taken together, reducing biologically available IL-6 seems to be a common effect of several antirheumatic drugs.

Altogether 18 circulating proteins were downregulated in patients treated with tofacitinib. Of these proteins, 14 are involved in the cellular responses to cytokine stimulation and 13 in the inflammatory responses (Table 3). In addition to IL-6, the function of which in the pathogenesis of RA is well documented [28], the majority of these proteins, such as IL-7, IL-12B, TNFSF14, chemokines, and MMP-1, are likely to promote the inflammatory and destructive processes in RA [1, 2]. Hence, their decreased expression may contribute to the clinical efficacy of tofacitinib. However, also the prototypic anti-inflammatory cytokine IL-10 [29] was decreased during tofacitinib treatment. It may be noted that the percentages of IL-10^+^ B cells after collagen epitope stimulation were shown to be reduced in RA patients and that tofacitinib treatment partially prevented this IL-10 downmodulation [30], indicating that tofacitinib may also function to promote IL-10 expression in certain situations. In healthy controls, instead, the percentages of IL-10^+^ B cells following collagen epitope stimulation slightly increased [30], highlighting the fact that studying the effects of tofacitinib in healthy controls does not necessarily stand for RA patients. Likewise, it has been observed that JAK inhibition using tofacitinib or baricitinib prevents reactive oxygen species (ROS) release from *in vitro* primed healthy control neutrophils but had no effect on ROS production by RA neutrophils [31].

The finding that plasma levels of three proteins, i.e. CX3CL1, MMP-10, and Flt3L, increased along with tofacitinib use may seem surprising. Indeed, contradicting our current results, a decline in CX3CL1 plasma levels has been seen in RA patients as a response to treatment with TNF inhibitors or a biologic drug abatacept, a fusion protein preventing T cell activation [32, 33]. Notably, the membrane-bound form of CX3CL1 is an adhesion molecule enhancing leukocyte adhesion and migration to the inflammatory foci, whereas its cleaved, soluble form acts as a chemokine for leukocytes [34]. Thus, an increase in the cleaved form at the expense of the intact molecule may indicate less effective adhesion but still possess proinflammatory potential. MMP-10 is present in synovial fluid and joint tissues of arthritis patients and potentiates cartilage collagenolysis [35]. However, MMP-10 may also be involved in the repair or healing of several tissue types [36, 37]. Flt3L acts as a differentiation factor for dendritic cells and is enriched in the serum and synovial fluid of RA patients, and has been suggested to progress arthritis [38, 39]. On the other hand, Flt3L signaling seems capable to control the formation of osteoclasts [40] and helps in maintaining the balance between protective regulatory T cells and pathogenic Th17 cells [39, 40]. Taken together, the effects of CX3CL1, MMP-10, and Flt3L can be diverse, and it may depend on the immunological context if a decrease or increase in their level is more favorable in arthritis.

There are several possible mechanisms on how tofacitinib may modulate plasma protein levels. First, the self-evident action of tofacitinib is the inhibition of JAK1 and JAK3, and to a slightly lesser extent JAK2, thus restraining cytokine-induced signaling to STATs and subsequent gene transcription [8, 9]. By studying circulating leukocytes of the same patients as in the current study, we recently demonstrated that tofacitinib suppresses both basal and cytokine-induced phosphorylation of STATs *in vivo* [21]. IL-6 expression is conventionally considered to be mediated via non-JAK/STAT signaling pathways, the foremost nuclear factor kappa light chain enhancer of activated B cells [28], and hence, the effect of tofacitinib on IL-6 production may be indirect. However, according to the novel data, IL-6 production can also be mediated via JAK/STAT3 [41], suggesting that tofacitinib may also directly contribute to the >60% decrease in IL-6 levels we observed.

Second, recent findings indicate that tofacitinib can also regulate cellular functions by affecting the levels of certain microRNAs [42]. Furthermore, novel results suggest that the effects of tofacitinib are associated with the functions of at least some histone deacetylases [43]. Disordered epigenetic mechanisms have been described in RA, and different epigenetic regulation patterns for various MMPs have been found in RA synovial fibroblasts [44]. Whether epigenetic mechanisms connecting tofacitinib and plasma protein expression exist, their exact molecular nature is to be elucidated.

Third, it should be noted that the production of the four plasma proteins that were most strongly reduced by tofacitinib in the current study is partly intertwined. For example, CXCL1 is one factor promoting IL-6 expression in RA synovial fibroblasts [45], and IL-6 increases MMP-1 expression in human chondrocytes [46]. In addition, in epithelial cells knockdown of AXIN1 significantly reduces lipopolysaccharide-induced IL-6 production [47]. Moreover, those 21 proteins that showed statistically significant and marked change by tofacitinib also have either known or predicted protein–protein interactions with each other as we illustrated using STRING analysis. These interactions can be direct physical or indirect functional associations. The possible multiplicative effects on the (near-by) members of the interaction network have to be considered, for example, when designing novel molecularly targeted treatments for RA.

Furthermore, it is possible that the decrease in RA disease activity itself during tofacitinib treatment is associated with the downregulation of inflammation-associated plasma proteins. In fact, when studying the levels of the 21 proteins markedly affected by tofacitinib use, we found that DAS28 improvement correlated with the decrease in the levels of two proteins, namely IL-6 and TNFSF14. Regarding that IL-6 is the crucial cytokine causing phosphorylation of the transcription factor signal transducer and activator of transcription (STAT3), the current finding is also well in line with what we recently found in the same patient population: the decrease in constitutive STAT3 phosphorylation levels in monocytes and CD4+ T cells correlated with DAS28 improvement during 3-month tofacitinib treatment [21]. Interestingly, TNFSF14 is emerging to be a potent contributor to joint injury, as it both promotes survival and activation of synovial fibroblasts in RA [48] and induces osteoclast formation and bone loss [49]. Hence, reduction of TNFSF14 levels might be involved in the structural protection of joints that accompany tofacitinib use.

We also examined if baseline DAS28 correlated with the levels of the 21 proteins markedly affected by tofacitinib use, and vice versa, if the plasma protein levels at baseline could predict treatment response to tofacitinib (determined by DAS28 improvement in 3 months). There were no associations between DAS28 at baseline and the baseline protein levels or the changes in the protein levels, suggesting that whereas DAS28 describes the extent of joint inflammation, the levels of circulating proteins may primarily reflect the systemic inflammatory processes. On the contrary, we found that high baseline IL-6 levels are associated with good treatment response, which is again in accordance with our previous findings demonstrating that constitutive STAT3 phosphorylation also associates with treatment response to tofacitinib [21]. Interestingly, while we previously noticed that STAT3 phosphorylation of circulating leukocytes was also associated with a good response to csDMARDs in recent-onset RA, plasma IL-6 levels were not [4]. Likewise, it has been reported that IL-6 levels cannot predict the treatment efficacy of MTX and prednisone in RA patients [25]. In contrast, a weak association between serum IL-6 levels and treatment response to tocilizumab has been demonstrated [50]. Nevertheless, it seems plausible that by suppressing both IL-6 signaling and production, tofacitinib counteracts this cytokine very effectively, which may be most discernible when the IL-6 levels are initially high.

DNER and CCL11 levels showed a negative correlation to treatment response. In patients with osteoarthritis, the gene of DNER was up-regulated in the affected cartilage [51]. However, little is known about the role of DNER in RA. CCL11 promotes leukocyte migration in RA, and serum and synovial fluid levels of CCL11 are elevated in RA patients [52]. OSM was among the proteins downregulated by tofacitinib use, but its plasma levels were not associated with the treatment response. Interestingly, high levels of circulating OSM have repeatedly been reported in patients with inflammatory bowel disease that does not respond to TNF inhibitors [53, 54]. It remains to be elucidated whether OSM could be a potential biomarker for predicting response to anti-TNF therapy also in RA. In general, a worthy approach, implemented also on the basis of our current results, would be using a small panel of potential markers for predicting treatment response to tofacitinib or other medications in RA.

There are several strengths in the current study: well-characterized RA patients from a prospective study are included, the *in vivo* setting is likely to reveal the real physiological effects of tofacitinib, and the number of simultaneously studied proteins is high, which enables the discovery of the effects of tofacitinib on novel proteins. A limitation is the relatively small size of the cohort, which allows only predictive conclusions on the association between plasma proteins and treatment response to tofacitinib. In particular, stringent adjustments for multiple testing and analysis of the data with respect to EULAR response criteria were not feasible in this setting.

To conclude, the current study revealed a comprehensive pattern of how tofacitinib treatment affects the levels of plasma proteins in patients with active csDMARD-irresponsive RA. These effects of tofacitinib were somewhat different from those previously described in *in vitro* studies or in animal models, highlighting the importance of analyzing protein expression in RA patients treated with tofacitinib. Most of the circulating proteins were downregulated, which may contribute to the clinical efficacy of tofacitinib since these proteins predominantly possess proinflammatory characteristics. Finally, due to the lack of biomarkers to predict treatment response to tofacitinib or other JAK inhibitors, the association of certain plasma proteins, such as IL-6, and treatment response to tofacitinib warrants further studies.

## Supplementary Material

uxac085_suppl_Supplementary_Figure_S1

uxac085_suppl_Supplementary_Figure_S2

uxac085_suppl_Supplementary_Tables

## Data Availability

Data is available from the authors on reasonable request.

## Abbreviations

CCL2: C-C motif chemokine ligand 2
CX3CL1: C-X3-C motif chemokine ligand 1
CXCL9: C-X-C motif chemokine ligand 9
csDMARD: conventional synthetic disease-modifying antirheumatic drug
DAS28-CRP(4): disease activity score for 28 joints based on C-reactive protein level
DAS28: 28-joint disease activity score
DNER: delta and notch-like epidermal growth factor-related Receptor
EDTA: ethylene diamine tetra acetic acid
Flt3L: FMS-like tyrosine kinase 3 ligand
IFN: interferon
IL-1: interleukin-1
IL-6: interleukin-6
JAK: Janus Kinase
LOD: limit of detection
MMP: matrix metalloproteinase
MTX: methotrexate
NPX: normalized protein expression
OSM: oncostatin M
RA: rheumatoid arthritis
STAT: signal transducer and activator of transcription
STRING: search tool for the retrieval of interacting genes/proteins
TNF: tumor necrosis factor-α
TNFSF14: tumor necrosis factor superfamily member 14.

## Clinical Trial Registration

This clinical trial has been registered in the EudraCT database, EudraCT number 2017-002753-11.
