# Supplementary figures and images for: Tofacitinib treatment modulates the levels of several inflammation-related plasma proteins in rheumatoid arthritis and baseline levels of soluble biomarkers associate with the treatment response
Source: Clin Exp Immunol. 2022 Sep 17;210(2):141–50. doi: 10.1093/cei/uxac085 (PMC9750823; doi:10.1093/cei/uxac085)

## Slide 1
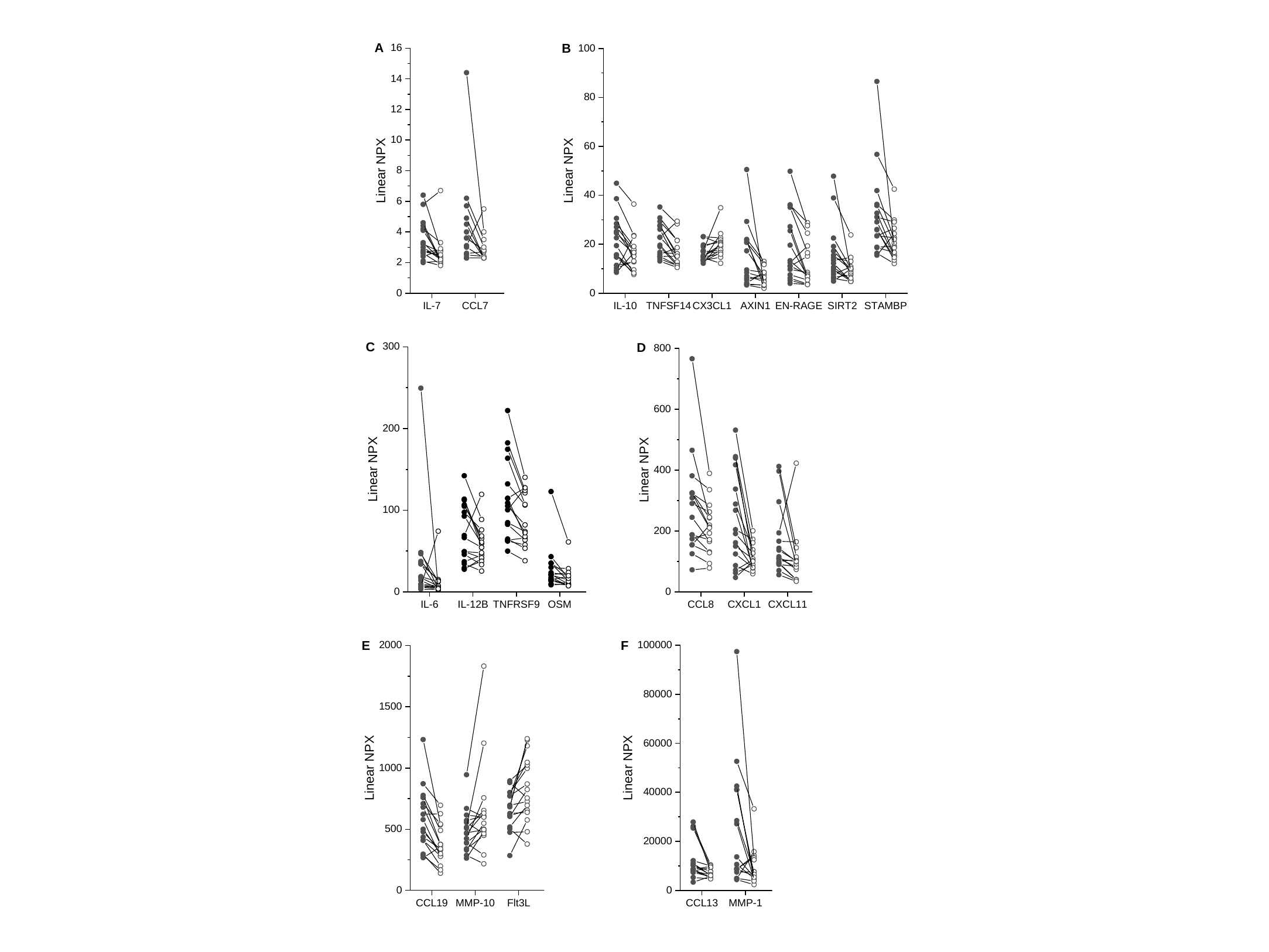

Supplement: uxac085_suppl_Supplementary_Figure_S1 [file uxac085_suppl_supplementary_figure_s1.pptx]

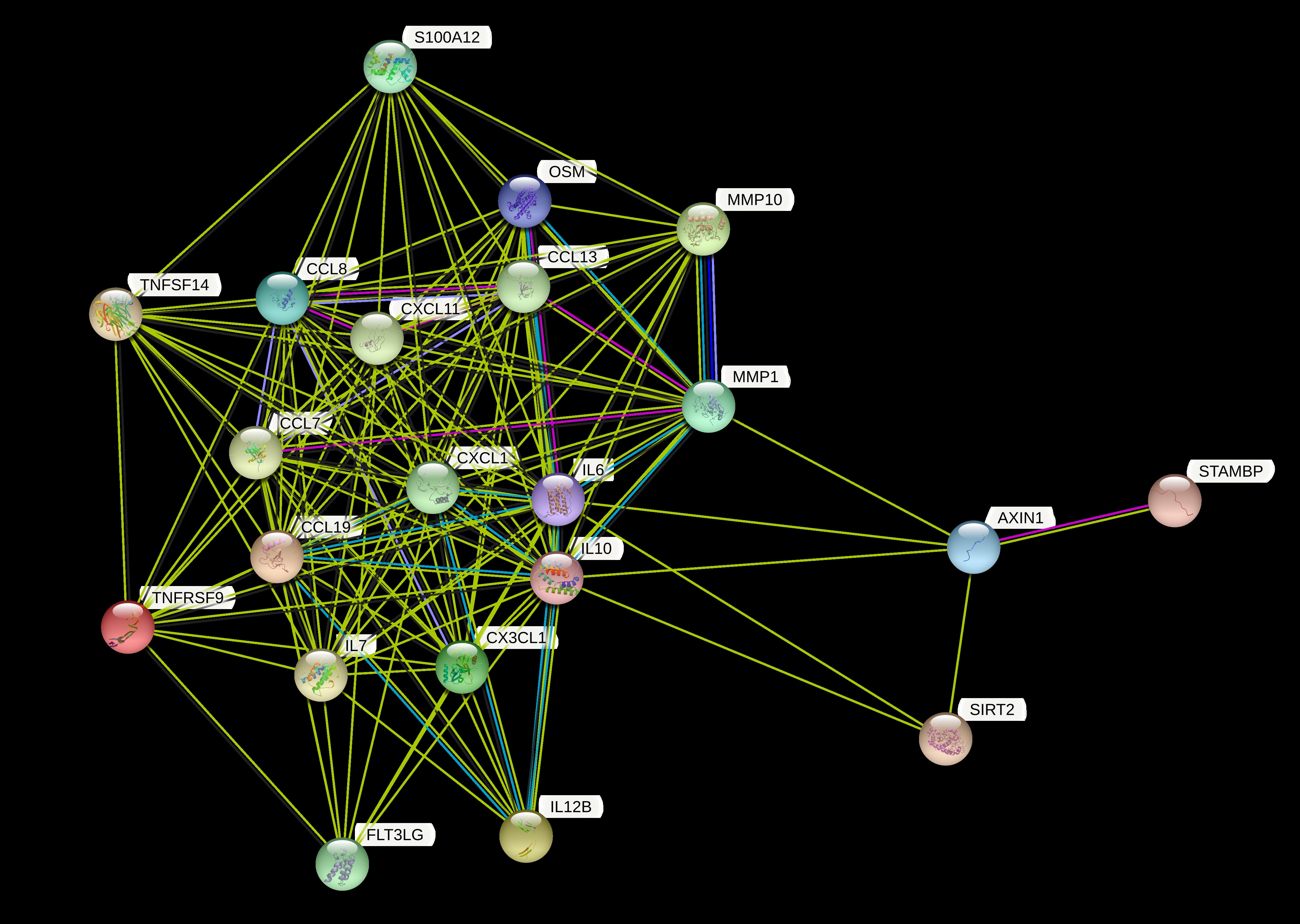

Supplement: uxac085_suppl_Supplementary_Figure_S2 [file uxac085_suppl_supplementary_figure_s2.jpeg]
