# Supplementary material for: Tofacitinib treatment modulates the levels of several inflammation-related plasma proteins in rheumatoid arthritis and baseline levels of soluble biomarkers associate with the treatment response
Source: Clin Exp Immunol. 2022 Sep 17;210(2):141–50. doi: 10.1093/cei/uxac085 (PMC9750823; doi:10.1093/cei/uxac085)
Supplement: uxac085_suppl_Supplementary_Tables [file uxac085_suppl_supplementary_tables.docx]

| Supplementary Table 1: Proteins analyzed in the proximity extension assay. | | | | | | | | | | | |
| --- | --- | --- | --- | --- | --- | --- | --- | --- | --- | --- | --- |
|  |  |  |  |  |  |  |  |  |  |  |  |
| Proteins with more than 25% values under limit of detection and omitted from further analyses | | | Proteins without statistically significant change during tofacitinib treatment (p>0.05) | | | Proteins with statistically significant (p<0.05) but less than 20% decrease | | | Proteins with statistically significant (p<0.05) and minimum 20% decrease | | |
|  |  |  |  |  |  |  |  |  |  |  |  |
| IL-1 alpha |  |  | IL-8 |  |  | DNER |  |  | IL-6 |  |  |
| IL-2 |  |  | IL-10RB |  |  | FGF-5 |  |  | IL-7 |  |  |
| IL-2RB |  |  | IL-17A |  |  | uPA |  |  | IL-10 |  |  |
| IL-4 |  |  | IL-17C |  |  | SCF |  |  | IL-12B |  |  |
| IL-5 |  |  | IL-18 |  |  |  |  |  | TNFSF14 |  |  |
| IL-10RA |  |  | IL-18R1 |  |  | Proteins with statistically significant (p<0.05) but less than 20% increase | | | TNFRSF9 |  |  |
| IL-13 |  |  | IFN-gamma |  |  |  |  |  | CCL7 |  |  |
| IL-20 |  |  | TNF |  |  |  |  |  | CCL8 |  |  |
| IL-20RA |  |  | CCL2 |  |  | CD5 |  |  | CCL13 |  |  |
| IL-22 RA1 |  |  | CCL3 |  |  | PD-L1 |  |  | CCL19 |  |  |
| IL-24 |  |  | CCL4 |  |  | IL-15RA |  |  | CXCL1 |  |  |
| IL-33 |  |  | CCL11 |  |  | LT-α |  |  | CXCL11 |  |  |
| ARTN |  |  | CCL20 |  |  | CD8A |  |  | MMP-1 |  |  |
| Beta-NGF |  |  | CCL23 |  |  | HGF |  |  | AXIN1 |  |  |
| LIF |  |  | CCL25 |  |  | CSF-1 |  |  | EN-RAGE |  |  |
| NRTN |  |  | CCL28 |  |  |  |  |  | OSM |  |  |
| TSLP |  |  | CXCL5 |  |  |  |  |  | SIRT2 |  |  |
|  |  |  | CXCL6 |  |  |  |  |  | STAMBP |  |  |
|  |  |  | CXCL9 |  |  |  |  |  |  |  |  |
|  |  |  | CXCL10 |  |  |  |  |  | Proteins with statistically significant (p<0.05) and minimum 20% increase | | |
|  |  |  | CDCP1 |  |  |  |  |  |  |  |  |
|  |  |  | CD6 |  |  |  |  |  |  |  |  |
|  |  |  | CD40 |  |  |  |  |  | CX3CL1 |  |  |
|  |  |  | CD244 |  |  |  |  |  | MMP-10 |  |  |
|  |  |  | 4E-BP1 |  |  |  |  |  | Flt3L |  |  |
|  |  |  | ADA |  |  |  |  |  |  |  |  |
|  |  |  | CASP-8 |  |  |  |  |  |  |  |  |
|  |  |  | CST5 |  |  |  |  |  |  |  |  |
|  |  |  | FGF-19 |  |  |  |  |  |  |  |  |
|  |  |  | FGF-21 |  |  |  |  |  |  |  |  |
|  |  |  | FGF-23 |  |  |  |  |  |  |  |  |
|  |  |  | GDNF |  |  |  |  |  |  |  |  |
|  |  |  | LAP TGF-beta-1 |  |  |  |  |  |  |  |  |
|  |  |  | LIF-R |  |  |  |  |  |  |  |  |
|  |  |  | NT-3 |  |  |  |  |  |  |  |  |
|  |  |  | OPG |  |  |  |  |  |  |  |  |
|  |  |  | SLAMF1 |  |  |  |  |  |  |  |  |
|  |  |  | ST1A1 |  |  |  |  |  |  |  |  |
|  |  |  | TGF-alpha |  |  |  |  |  |  |  |  |
|  |  |  | TRAIL |  |  |  |  |  |  |  |  |
|  |  |  | TRANCE |  |  |  |  |  |  |  |  |
|  |  |  | TWEAK |  |  |  |  |  |  |  |  |
|  |  |  | VEGFA |  |  |  |  |  |  |  |  |
| Interleukin (IL); artemin (ARTN); beta-nerve growth factor (Beta-NGF); leukemia inhibitory factor (LIF); neurturin (NRTN); thymic stromal lymphopoietin (TSLP); interferon (IFN); tumor necrosis factor (TNF); C-C motif chemokine (CCL); C-X-C motif chemokine (CXCL); CUB domain-containing protein 1 (CDCP1); cluster of differentiation (CD); eukaryotic translation initiation factor 4E-binding protein (4E-BP1); adenosine deaminase (ADA); caspase-8 (CASP-8); cystatin D (CST5); fibroblast growth factor (FGF); glial cell line-derived neurotrophic factor (GDNF); latency-associated peptide transforming growth factor beta-1 (LAP TGF-beta-1); leukemia inhibitory factor receptor (LIF-R); neutrophin-3 (NT-3); osteoprotegerin (OPG); signaling lymphocytic activator molecule (SLAMF1); transforming growth factor alpha (TGF-alpha); TNF-related apoptosis-inducing ligand (TRAIL); TNF-related activation-induced cytokine (TRANCE); tumor necrosis factor (Ligand) superfamily, member 12 (TWEAK); vascular endothelial growth factor A (VEGF-A); delta and Notch-like epidermal growth factor-related receptor (DNER); urokinase-type plasminogen activator (uPA); stem cell factor (SCF); programmed cell death 1 ligand 1 (PD-L1); Lymphotoxin-alpha (LT-α); hepatocyte growth factor (HGF); macrophage colony-stimulating factor (CSF-1); tumor necrosis factor superfamily member 14 (TNFSF14); tumor necrosis factor receptor superfamily member 9 (TNFRSF9); matrix metalloproteinase (MMP); S100 calcium binding protein A12 (EN-RAGE); Oncostatin M (OSM); NAD-dependent deacetylase sirtuin 2 (SIRT2); STAM-binding protein (STAMBP); C-X3-C motif chemokine ligand (CX3CL); FMS-like tyrosine kinase 3 ligand (Flt3L). | | | | | | | | | | | |

| Supplementary table 2: Correlations between DAS28-4[CRP] at baseline and plasma protein levels at baseline (left panel) or changes in plasma protein levels during 3-month tofacitinib treatment (right panel) in patients with rheumatoid arthritis. Correlations were analyzed only for proteins that showed statistically significant (p<0.05) and minimum 20% difference by tofacitinib treatment. | | | | | | | |
| --- | --- | --- | --- | --- | --- | --- | --- |
|  |  |  |  |  |  |  |  |
|  |  | Level at baseline | |  | Change during treatment | |  |
|  |  | r | p |  | r | p |  |
| IL-6 |  | 0.236 (-0.215 to 0.602) | 0.380 |  | -0.181 (-0.562 to 0.288) | 0.502 |  |
| IL-7 |  | -0.074 (-0.672 to 0.644) | 0.786 |  | 0.384 (-0.240 to 0.804) | 0.142 |  |
| IL-10 |  | -0.337 (-0.692 to 0.190) | 0.201 |  | 0.483 (-0.100 to 0.824) | 0.058 |  |
| IL-12B |  | -0.192 (-0.817 to 0.413) | 0.475 |  | -0.192 (-0.728 to 0.353) | 0.475 |  |
| TNFSF14 |  | -0.035 (-0.567 to 0.561) | 0.897 |  | 0.090 (-0.379 to 0.535) | 0.741 |  |
| TNFRSF9 |  | -0.121 (-0.687 to 0.505) | 0.656 |  | 0.189 (-0.319 to 0.626) | 0.484 |  |
| CCL7 |  | 0.265 (-0.285 to 0.753) | 0.321 |  | 0.015 (-0.462 to 0.502) | 0.956 |  |
| CCL8 |  | 0.091 (-0.666 to 0.863) | 0.737 |  | -0.065 (-0.694 to 0.460) | 0.812 |  |
| CCL13 |  | -0.081 (-0.697 to 0.550) | 0.766 |  | 0.239 (-0.287 to 0.650) | 0.374 |  |
| CCL19 |  | 0.056 (-0.568 to 0.598) | 0.837 |  | 0.099 (-0.417 to 0.548) | 0.716 |  |
| CXCL1 |  | -0.087 (-0.598 to 0.470) | 0.749 |  | 0.155 (-0.365 to 0.700) | 0.567 |  |
| CXCL11 |  | -0.106 (-0.817 to 0.671) | 0.696 |  | 0.306 (-0.162 to 0.689) | 0.249 |  |
| CX3CL1 |  | 0.130 (-0.343 to 0.531) | 0.632 |  | -0.124 (-0.628 to 0.454) | 0.648 |  |
| MMP-1 |  | 0.016 (-0.682 to 0.693) | 0.953 |  | 0.068 (-0.502 to 0.580) | 0.803 |  |
| MMP-10 |  | 0.090 (-0.514 to 0.616) | 0.741 |  | 0.105 (-0.398 to 0.531) | 0.700 |  |
| AXIN1 |  | 0.367 (-0.158 to 0.728) | 0.162 |  | -0.136 (-0.588 to 0.481) | 0.615 |  |
| EN-RAGE |  | -0.027 (-0.549 to 0.534) | 0.922 |  | -0.069 (-0.577 to 0.479) | 0.799 |  |
| Flt3L |  | 0.225 (-0.380 to 0.781) | 0.401 |  | 0.225 (-0.420 to 0.772) | 0.401 |  |
| OSM |  | -0.222 (-0.695 to 0.421) | 0.408 |  | 0.171 (-0.360 to 0.614) | 0.527 |  |
| SIRT2 |  | 0.077 (-0.494 to 0.544) | 0.778 |  | 0.119 (-0.293 to 0.506) | 0.662 |  |
| STAMBP |  | 0.067 (-0.540 to 0.575) | 0.805 |  | 0.240 (-0.190 to 0.619) | 0.370 |  |
| Spearman correlation coefficients (r) and confidence intervals (CI) calculated with bootstrapping are shown. Composite Disease Activity Score for 28 joints based on C-reactive protein level (DAS28-4[CRP]); conventional synthetic disease-modifying antirheumatic drug (csDMARD); interleukin (IL); Tumor necrosis factor superfamily member 14 (TNFSF14); tumor necrosis factor receptor superfamily member 9 (TNFRSF9); C-C motif chemokine (CCL); C-X-C motif chemokine (CXCL); C-X3-C motif chemokine (CX3CL); matrix metalloproteinase (MMP); S100 calcium binding protein A12 (EN-RAGE); FMS-like tyrosine kinase 3 ligand (Flt3L); oncostatin M (OSM); NAD-dependent deacetylase sirtuin 2 (SIRT2); STAM-binding protein (STAMBP). | | | | | | | |
